# Applying machine learning to the pharmacokinetic modeling of cyclosporine in adult renal transplant recipients: a multi-method comparison

**DOI:** 10.3389/fphar.2022.1016399

**Published:** 2022-10-24

**Authors:** Junjun Mao, Yuhao Chen, Luyang Xu, Weihuang Chen, Biwen Chen, Zhuo Fang, Weiwei Qin, Mingkang Zhong

**Affiliations:** ^1^ Department of Pharmacy, Huashan Hospital, Fudan University, Shanghai, China; ^2^ Department of Data and Analytics, WuXi Diagnostics Innovation Research Institute, Shanghai, China

**Keywords:** cyclosporine, renal transplantation, machine learning, population pharmacokinetic model, artificial neural network, predictive performance

## Abstract

**Objective:** The aim of this study was to identify the important factors affecting cyclosporine (CsA) blood concentration and estimate CsA concentration using seven different machine learning (ML) algorithms. We also assessed the predictability of established ML models and previously built population pharmacokinetic (popPK) model. Finally, the most suitable ML model and popPK model to guide precision dosing were determined.

**Methods:** In total, 3,407 whole-blood trough and peak concentrations of CsA were obtained from 183 patients who underwent initial renal transplantation. These samples were divided into model-building and evaluation sets. The model-building set was analyzed using seven different ML algorithms. The effects of potential covariates were evaluated using the least absolute shrinkage and selection operator algorithms. A separate evaluation set was used to assess the ability of all models to predict CsA blood concentration. R squared (*R* ^2^) scores, median prediction error (MDPE), median absolute prediction error (MAPE), and the percentages of PE within 20% (F_20_) and 30% (F_30_) were calculated to assess the predictive performance of these models. In addition, previously built popPK model was included for comparison.

**Results:** Sixteen variables were selected as important covariates. Among ML models, the predictive performance of nonlinear-based ML models was superior to that of linear regression (MDPE: 3.27%, MAPE: 34.21%, F_20_: 30.63%, F_30_: 45.03%, *R* ^2^ score: 0.68). The ML model built with the artificial neural network algorithm was considered the most suitable (MDPE: −0.039%, MAPE: 25.60%, F_20_: 39.35%, F_30_: 56.46%, *R* ^2^ score: 0.75). Its performance was superior to that of the previously built popPK model (MDPE: 5.26%, MAPE: 29.22%, F_20_: 33.94%, F_30_: 51.22%, *R* ^2^ score: 0.68). Furthermore, the application of the most suitable model and the popPK model in clinic showed that most dose regimen recommendations were reasonable.

**Conclusion:** The performance of these ML models indicate that a nonlinear relationship for covariates may help to improve model predictability. These results might facilitate the application of ML models in clinic, especially for patients with unstable status or during initial dose optimization.

## 1 Introduction

Cyclosporine (CsA) is a potent calcineurin inhibitor widely used to prevent allograft rejection in solid organ transplantation (Fahr, 1993; Meier-Kriesche et al., 2006). Given its narrow therapeutic index and large inter- and intra-individual pharmacokinetic/pharmacodynamic (PK/PD) variabilities, conducting routine therapeutic drug monitoring (TDM) is essential to optimize CsA dosage regimens and minimize adverse effects (Shaw et al., 1987).

Currently, the pre-dose (C_0_) and 2 h post-dose concentrations (C_2_) of CsA are conventionally monitored during clinical follow-up. Population PK (popPK) models combined with maximum a *posteriori* Bayesian estimators (MAP-BE) are used to establish a dose titration guide, which is more precise than the prescription depending only on the personal experiences of physicians (Asberg et al., 2010). With the advances in computer technology, precision dosing based on pharmacogenetics and PK/PD models has been suggested to improve patient care (Mizuno et al., 2020).

Based on the compartmental model theory, popPK models can describe the drug PK behavior of individuals by applying statistical mixed effect methods with PK parameters (Sheiner et al., 1977; Sheiner and Beal, 1980). In a previous study, we attempted to identify factors that explain the variability of the CsA PK properties and characterize the time-varying clearance (CL/*F*) by comprehensively analyzing the CsA PK process using popPK modeling (Mao et al., 2021). Although more theoretical mechanisms were considered to improve model transferability, describing the drug *in vivo* process was challenging in patients with unstable conditions. In addition, in the context of the rapidly changing clinical status and inflammatory state of renal transplant recipients, the assumptions of the structural model may be inaccurate or overly simplistic.

Contrary to PK-based approaches, which aims to describe the physiological phenomena involved in the drug *in vivo* process and its variability between individuals, machine learning (ML) models are accuracy-centered, data-driven approaches that eliminate the need for mechanistic assumptions (Badillo et al., 2020). A traditional ML algorithm can be something as simple as linear regression. They use a variety of statistical techniques to interpret the existing data without having to be programmed explicitly. Moreover, artificial neural network (ANN) is a specialized subset of ML algorithms, which describes algorithms that analyze data with a logical structure similar to how a human would draw conclusions. Inspired by the biological neural network of the human brain, ANN uses a layered structure of algorithms to learn a set of complex relationships between the variables, leading to a learning process that is far more capable than that of traditional ML algorithms (Popescu et al., 2009).

As ML models can capture the complex relationships between variables and analyze high-dimensional data in clinical practice, these have been used in clinical pharmacology in recent years. For example, Woillard *et al.* used ML models to estimate the glomerular filtration rate of intensive care unit patients, based on sparse iohexol PK data (Woillard et al., 2021c). This approach was also used to predict the exposure of tacrolimus (Woillard et al., 2021b) and mycophenolic acid (Woillard et al., 2021a). Moreover, Tang *et al.* combined popPK and ML models to improve the prediction of individual clearance of renally cleared drugs in neonates (Tang et al., 2021).

According to our previous study, the incorporation of nonlinear kinetics during the modeling process can improve the predictive performance of popPK models for CsA in adult renal transplant recipients (Mao et al., 2020). Furthermore, rather than defining a structural model to describe the observed data, ML models use algorithmic modeling of multiple variables linked by complex interactions to obtain nonlinear relationships that predict clinical outcomes with high accuracy (Badillo et al., 2020; Gautier et al., 2021). In pharmacokinetics, these methods can estimate clearance through characteristics of the patient, such as demographic characteristics, pathophysiological indexes, disease status, and associated medications.

In this study, we aimed to identify the important covariates of the CsA concentration based on retrospective data and estimate the CsA concentration using multiple ML models. We compared the predictions obtained in this study with those of the previously developed popPK model (Mao et al., 2021). Then, the most suitable ML and popPK models were applied to guide personalized medicine.

## 2 Materials and methods

### 2.1 Study group and data collection

We recruited 183 renal transplant patients (122 males and 61 females) at Huashan Hospital. Patients were administered combined immunosuppressive therapy, including a CsA microemulsion (Neoral; Novartis Pharma Schweiz AG, Emberbach, Germany), mycophenolate mofetil (MMF; CellCept; Roche Pharma Ltd., Shanghai, China), and steroids. The detailed therapeutic regimens are described in Supplementary Text S1.

The inclusion criteria were as follows: patients 1) aged ≥18 years, 2) who had received their first allograft renal transplantation and 3) who had received a CsA-based triple immunosuppressive regimen. The exclusion criteria were as follows: those who 1) received the conventional, oral formulation of CsA, 2) underwent dialysis treatment, and 3) had missing covariate data required for analyses.

The study protocols were approved by the Ethics Committee of Huashan Hospital and conducted in accordance with the Declaration of Helsinki. All patients provided written informed consent and agreed to the anonymous use of their samples in this study.

We retrospectively collected PK samples of CsA C_0_ and C_2_ from the enrolled patients during follow-up TDM. All samples were stored at −20°C for CsA concentration determination, biochemical assay, and pharmacogenetic tests. Details regarding to the determination of CsA concentration and genotyping are presented in Supplementary Text S2 and S3 respectively.

### 2.2 Machine learning model development

Seven ML models including six traditional models and an ANN model were applied to describe the relationship between variables and CsA concentration.

Our study comprised the following steps:

Step 1: Covariate selection was performed using the least absolute shrinkage and selection operator (LASSO).

Step 2: Seven ML algorithms were used to construct the prediction models.

Step 3: The predictability of the ML models and that of the previously built popPK model were evaluated.

Step 4: The most suitable ML model and the previously built popPK model were used to guide precision dosing.

**FIGURE 1:**
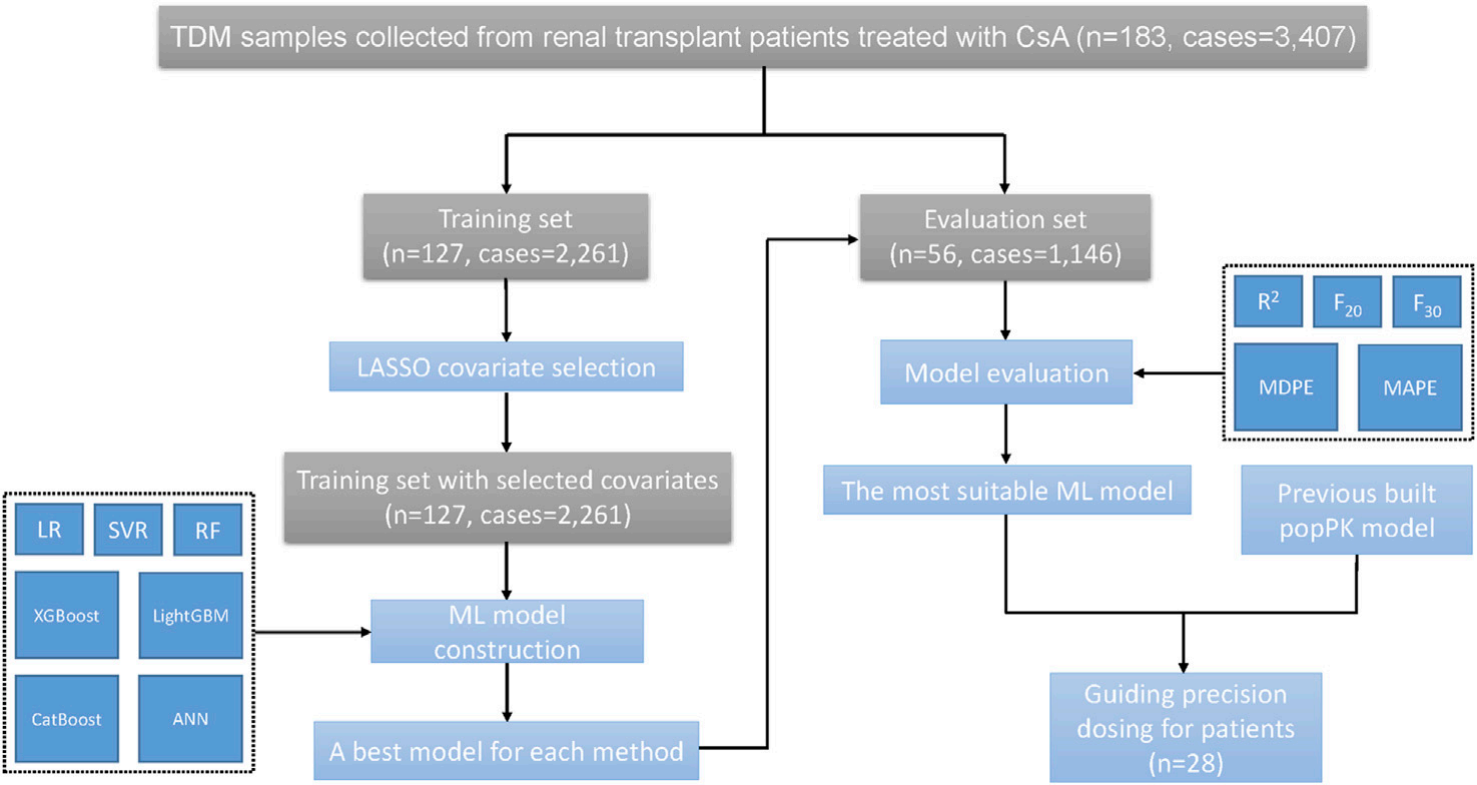
The flowchart of study procedures.

The flowchart of these procedures is shown in Figure 1.

#### 2.2.1 Data preparation and covariate selection

The patients were divided into training and evaluation sets, as described in our previous study (Mao et al., 2021). The samples collected were used for model construction and evaluation. For each CsA concentration, the two latest CsA doses before measurement were identified as the major predictors. Furthermore, 57 other variables (e.g., demographics, pathophysiological characteristics, concomitant medications, and pharmacogenomic information) were identified as covariates. An integrated abbreviation list of all variables and their corresponding explanations is provided in Supplementary Table S1.

After data separation, the training set included 127 patients with complete data of all variables, whereas the evaluation set had missing genetic information from 16 of 56 patients. The missing data were imputed using the genotype with the highest frequency among the remaining subjects. Numerical variables were scaled to 0–1. Covariate selection was conducted using LASSO (Alhamzawi and Ali, 2018).

LASSO was used to obtain the predictor subset that minimized the prediction error (PE) for the variables. LASSO applied a constraint on the model parameters by using a generalized linear model *via* penalized maximum likelihood, shrinking the regression coefficients of some variables toward zero. Variables with regression coefficients equal to zero were excluded from the model. The R software package glmnet was used for the LASSO analysis, and the model was evaluated using a 10-fold cross-validation procedure (Friedman et al., 2022).

Specifically, the training data were split into 10 independent folds of approximately equal size. The models were trained using nine folds of the data and then tested using the remaining fold; this procedure was repeated for each of the 10 training and testing fold combinations.

#### 2.2.2 Six traditional machine learning modeling

Along with the selected variables, six traditional ML algorithms, including linear regression (LR), support vector regression (SVR), random forest (RF), XGBoost, LightGBM, and CatBoost were used for model building (Mahabub, 2019). These ML models were implemented using the “scikit-learn” (sklearn) module in Python 3.6 (Pedregosa et al., 2011). Similarly, a 10-fold cross-validation procedure was performed for the parameter tuning and performance evaluation of each method in the training set. Root mean square error (RMSE) was used to select the best parameter combinations. The model built with the fine-tuned parameters was used as the final model for each method.

#### 2.2.3 Artificial neural network modeling

A multilayer perceptron (MLP) is a fully connected class of feedforward ANN. It consists of at least three layers of nodes: an input layer, a hidden layer, and an output layer (Popescu et al., 2009). The input layer receives the input signal to be processed. The required task, such as prediction or classification, is performed by the output layer. An arbitrary number of hidden layers that are placed in between the input and output layers are the true computational engine.

In this study, an MLP neural network model, which consisted of an input layer, two hidden layers, and an output layer, was constructed for CsA concentration prediction using the “keras” module in Python 3.6 (Gulli A, 2017). To obtain the best generalization performance, we induced a dropout layer behind each hidden layer and applied an early stop strategy to stop model learning before overfitting.

The patients in the training set were randomly divided into model building (including 70% of the patient samples) and model validation (including the remaining 30% of the patient samples) sets. The hyper-parameters, including the number of neurons in the hidden layer, activation function, dropout rate, and batch sizes, the values of which were used to control the learning process, were fine-tuned using the model building set and evaluated using the model validation set.

The mean squared error was used as the loss function metric, and Adam was used as the optimizer. The model with the highest *R* ^2^ value in the model validation set was selected as the most suitable model. Using all samples in the training set, the associated combination of hyper-parameters was used to construct the final model.

### 2.3 Model evaluation

Each model of different algorithms was validated using samples from an independent evaluation dataset. The coefficient of determination *R* ^2^ scores, prediction-based PE (Eq. 1), median prediction error (MDPE), and median absolute prediction error (MAPE) were used to compare the accuracy and precision of model predictive performance (Sheiner and Beal, 1981). *R* ^2^ is the squared correlation between predicted and observed CsA concentrations, with higher values indicating better predictability. The model with the highest *R* ^2^ values and the lowest MDPE and MAPE values was considered the most suitable model.

$$PE\;\left(\%\right)=\left(\frac{PRED-OBS}{OBS}\right)\times 100$$ (1)

The percentages of PE within 20% (F_20_) and 30% (F_30_) were used as the combination index of both accuracy and precision. Furthermore, the prediction performance of the ML models was compared with that of the previously developed popPK model in the same evaluation dataset (Mao et al., 2021).

The predictive performance of a candidate model was considered satisfactory given the following values: MDPE ≤ ± 15%, MAPE ≤30%, F_20_ > 35%, and F_30_ > 50% (Mao et al., 2018). Among all models, the one associated with the best prediction performance was selected as the most suitable ML model. The scatter plots of the predicted *versus* reference CsA concentrations and the distribution plots of percentage prediction errors were drawn for visualization.

### 2.4 Model application

The most suitable ML model and the previously built popPK model were used to guide precision dosing, and the dose regimens suggested by these two models were compared. Patients in the evaluation dataset with information on the early stages of transplantation were used for dosage adjustments. For each patient, we selected the first sample from postoperative day (POD) 10–15, based on our hypothesis that the concentrations of these samples were at steady state.

The most suitable ML and popPK models were used to optimize the initial dose of these patients. Target C_0_ values of 200–350 ng ml^−1^ and target C_2_ values of 1000–1500 ng ml^−1^ were suggested for the first month of CsA treatment (Shi and Yuan, 2016). For the ML model, a series of CsA dosages was input into the model to fit the upper and lower limits of recommended target C_0_ and C_2_, respectively. Then, the lower and upper limits of CsA dosage were recommended for each patient. Using the popPK model, we conducted Monte Carlo simulations as previously published (Mao et al., 2021). Time-concentration profiles were simulated based on 1000 hypothetical individuals. The C_0_/C_2_ value of the CsA doses was simulated from 50 mg q12h to 300 mg q12 h for each patient. The median and the 25th to 75th percentiles of a steady-state C_0_/C_2_ value were calculated, and the optimal dosing regimen was selected according to the target concentration. Finally, the rationality of the suggested dose regimens was assessed.

## 3 Results

### 3.1 Patients

Detailed demographics and clinical statistics are presented in Table 1. In total, 183 renal transplantation recipients were recruited for this study. Furthermore, 3,407 whole-blood CsA measurements were available, with 1,621 C_0_ and 1,786 C_2_. Concentrations below the lower quantification limit were not included in the analysis.

**TABLE 1:** Patients demographics used to develop and evaluate models.

Characteristics	Model development dataset	Model evaluation dataset
	Number or median (Range)	Number or median (Range)
No. of patients (Male/Female)^a^	127 (81/46)	56 (41/15)
No. of Samples (C_0_/C_2_)^b^	2261 (1081/1180)	1146 (541/605)
Age (years)	41 (19–60)	41 (18–58)
Height (cm)	168.0 (150.0–188.0)	170.0 (150.0–186.0)
Weight (kg)	60.0 (40.0–95.0)	61.0 (39.4–90.0)
Fat-free mass (kg)	46.4 (28.9–66.2)	50.3 (28.7–67.1)
Post-operation days	472 (1–5998)	111.5 (2–3942)
CsA daily dose (mg day^−1^)	275 (50–575)	250.0 (50–600)
Prednisolone dose (mg day^−1^)	20.0 (0–80)	7.5 (0–80)
C_0_ (ng ml^−1^)	157.9 (22.6–974.6)	123.6 (25.4–587.4)
C_2_ (ng ml^−1^)	805.2 (108.8–2572.8)	703.0 (34.6–2109.0)
Hematocrit (%)	31.1 (10.5–60.5)	34.7 (15.6–57.0)
Blood platelet (×10^9^ L^−1^)	174 (31–514)	192 (37–523)
Total Bilirubin (μmol L^−1^)	10.5 (1.0–168.9)	10.8 (1.7–48.3)
Alanine aminotransferase (U L^−1^)	32.1 (4.0–420.0)	19.0 (3.0–374.0)
Aspartate transferase (U L^−1^)	23.8 (5.0–383.0)	18.0 (1.0–279.0)
r-Glutamyl transpeptidase (U L^−1^)	20 (1–509)	20 (2–775)
Albumin (g L^−1^)	36.2 (20.0–52.0)	37.4 (22.0–51.0)
Total protein (g L^−1^)	63.2 (41.0–88.0)	67.0 (46.0–88.0)
Urea nitrogen (mmol L^−1^)	8.7 (2–54.1)	7.9 (2.9–44.8)
Serum creatinine (μmol L^−1^)	134.3 (14.0–1088.0)	107.0 (48.0–776.0)
Creatinine clearance (ml min^−1^)^c^	62.5 (6.2–360.7)	67.1 (6.2–182.6)
Concomitant medications^a^		
Felodipine	74	23
Nifedipine	46	18
Perdipine	8	7

C_0_, pre-dose concentration; C_2_, 2-h post-dose concentration.

^a^ Data are expressed as number of patients.

^b^ Data are expressed as number of samples.

^c^ Calculated following the Cockcroft-Gault formula: CLcr = [(140-Age (year)) ×WT (kg)]/(0.818×Scr (μmol L^−1^)) × (0.85 for female).

All observed genotypic distributions of CYP3A4*1G, CYP3A5*3, and *ABCB1* genetic polymorphisms were in accordance with the Hardy-Weinberg equilibrium (Supplementary Table S2). Only haplotypes with frequencies and patient proportions ≥8% were analyzed (Supplementary Table S3).

### 3.2 Covariate selection

Sixteen variables with non-zero coefficients and minimal prediction errors were selected using LASSO as the most important covariates (Supplementary Table S4), which were subsequently used for model construction with seven different ML algorithms: sampling time (C_0_ or C_2_), the two latest CsA doses before each sampling time, height, POD, white blood cell (WBC), hematocrit (HCT), blood platelet (PLT), total bilirubin (TBIL), r-glutamyl transpeptidase (rGT), urea nitrogen (UN), creatinine (CR), creatinine clearance rate (CLCR), acyclovir (ACI) use, norvasc (NOR) use, and MDR1 haplotypes CGC. Out of 16 covariates selected by LASSO, only three covariates (POD, HCT, and MDR1 haplotype CGC) were consistent with those of the previously built popPK model based on the same dataset (Mao et al., 2021).

### 3.3 Model construction and evaluation

**FIGURE 2:**
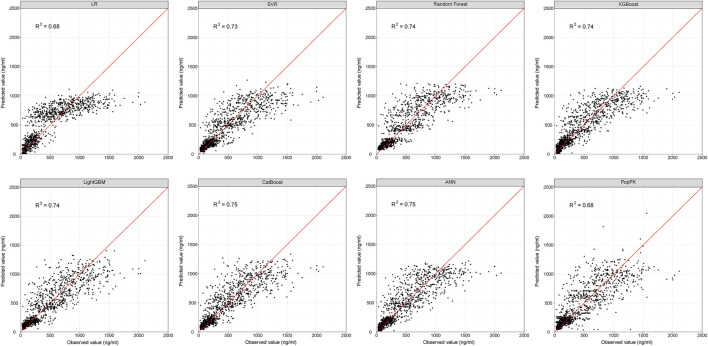
Scatter plots showing the reference and predicted cyclosporine concentrations from the evaluation dataset for ML and popPK models. Red line represents the reference line. ANN, artificial neural network; ML, machine learning; popPK, population pharmacokinetic model; *R* ^2^, the squared correlation between the predicted and observed concentrations; LR, linear regression; SVR, support vector regression.

The best-tuned parameters for six traditional ML models (i.e., LR, SVR, RF, XGBoost, LightGBM, and CatBoost) and the hyper-parameters selected for the ANN model are presented in Supplementary Table S5. The prediction performance of all models in the evaluation dataset is presented in Table 2. The previously developed popPK model was also included for comparison (Mao et al., 2021). The predicted CsA concentrations vs. observed concentrations for each method, along with the *R* ^2^ scores of all models, are presented in Figure 2.

**TABLE 2:** Predictive performance of seven ML models and previously built popPK model in the evaluation dataset.

Methods	MDPE	MAPE	F_20_	F_30_	*R* ^2^
LR	3.27	34.21	30.63	45.03	0.68
SVR	3.59	26.83	38.92	54.19	0.73
Random Forest	9.56	27.56	37.00	53.66	0.74
XGBoost	2.90	27.43	35.43	54.28	0.74
LightGBM	4.64	25.99	38.05	55.15	0.74
CatBoost	7.96	26.09	39.79	56.20	0.75
ANN	-0.039	25.60	39.35	56.46	0.75
popPK	5.26	29.22	33.94	51.22	0.68

ANN, artificial neural network; F_20_, F_30_, the percentages of prediction error within 20% and 30%, respectively; LR, linear regression; MAPE, median absolute prediction error; MDPE, median prediction error; popPK, population pharmacokinetic model; *R* ^2^, the squared correlation between the predicted and observed concentrations; SVR, support vector regression.

All ML models besides linear regression were developed based on nonlinear methods. The predictive performance of nonlinear-based ML models met the aforementioned criteria (i.e., MDPE ≤ ± 15%, MAPE ≤30%, F_20_ > 35% and F_30_ > 50%), except the linear regression model, which had an MDPE of 3.27%, MAPE of 34.21%, F_20_ of 30.63%, and F_30_ of 45.03%. This indicated that considering the nonlinear relationship of patient covariates may help improve model predictability.

**FIGURE 3:**
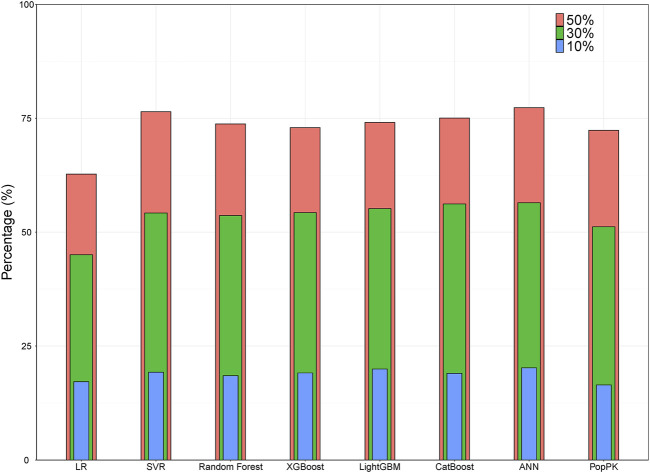
Bar plot showing the percentages of prediction error within 10%, 30%, and 50% for ML and popPK models. ANN, artificial neural network; ML, machine learning; popPK, population pharmacokinetic model; LR, linear regression; SVR, support vector regression.

With *R* ^2^ as the assessment metrics, the popPK model was slightly superior to the linear regression model but was inferior to other ML models. The percentages of samples with prediction errors within 10%, 30%, and 50% are shown in Figure 3. Among these models, the highest percentages were consistently achieved with the ANN model. Here, the predicted CsA concentrations within the prediction errors of 10%, 30%, and 50% were 20.24%, 56.46%, and 77.31%, respectively. In both accuracy and precision, the ANN model was considered the most suitable ML model with an MDPE of -0.039%, MAPE of 25.60%, F_20_ of 39.35%, F_30_ of 56.46%, and *R* ^2^ score of 0.75.

### 3.4 Model application

Twenty-eight patients from the evaluation dataset were selected for dosage adjustment. In C_0_, subtherapeutic and supratherapeutic CsA concentrations were observed in 53.6% and 3.6% of patients, respectively. In C_2_, subtherapeutic and supratherapeutic CsA concentrations were observed in 67.9% and 3.6% of patients, respectively. The median POD of these TDM values was 11, indicating the need for an initial dose design.

The results of the dosing regimen optimization conducted using the most suitable ML and popPK models are shown in Supplementary Table S6 and Supplementary Table S7. Most dose regimens suggested by the two models were reasonable, except for patients #192, #201, #812, and #909, whose concentrations were below the target ranges. Although the doses suggested by the ML model and popPK model were higher than the actual dosage. They were inconsistent with each other. The doses suggested by the ML model were higher than those suggested by the popPK model. However, for patient #169, whose concentrations were in the target ranges, a lower dose proposal was suggested by the ML model than by the popPK model and the actual dosage.

**FIGURE 4:**
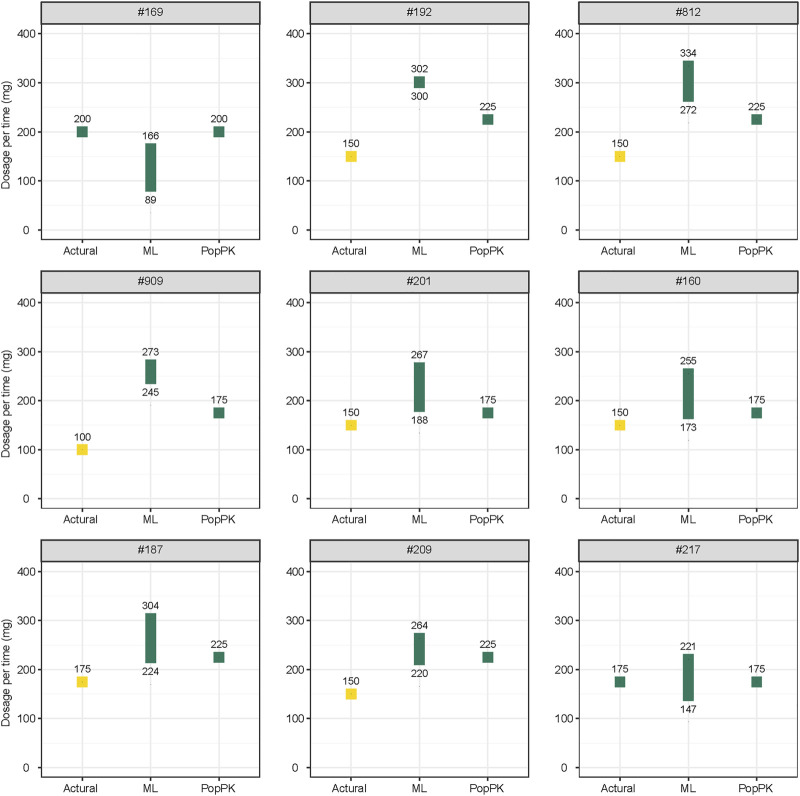
Comparison of the actual and optimal daily doses of cyclosporine recommended by the most suitable ML model and popPK models. Yellow plot indicates the concentration below the therapeutic windows; green plot indicates the concentration in the therapeutic windows. All doses are recommended twice daily. ML, machine learning; popPK, population pharmacokinetic model.

A comparison of the actual CsA dose, which was used based on the personal experiences of physicians, and optimal daily doses of CsA recommended by the most suitable ML and popPK models is shown in part in Figure 4, and the complete comparison for all patients is presented in Supplementary Figure S1.

## 4 Discussion

In this study, we systematically established seven ML models to predict blood trough and peak concentrations from CsA daily dose and other important variables selected using the LASSO algorithm. Thirteen out of 16 covariates compared to those of the previously built popPK model were newly identified.

LASSO is usually employed to get a quick idea of which covariates are important for predicting the outcome variable. It is unsuitable when the number of variables is greater than the number of observations and when many variables are correlated (Laura Freijeiro-González, 2022). The dataset we collected had a large number of observations, with mostly independent variables and a few collinear variables, which generally eliminates the limitation of LASSO. One advantage of LASSO is that it quickly incorporates a reduced set of variables, which are interpretable and reduce the complexity for the next step of model building.

Among these important covariates, the *MDR1* haplotype CGC is the only genetic factor. Allelic variations in exons 12 (1236C>T), 21 (2677G>T/A), and 26 (3435C>T) of the *MDR1* gene are associated with altered P-glycoprotein (P-gp) function (Kim, 2002), which contributes to the bioavailability of P-gp substrates, such as CsA (Zhang et al., 2008; Mao et al., 2021). Therefore, the *MDR1* haplotype was thought to be associated with CsA blood concentration. Based on our previous study, the CL/*F* of non-CGC haplotype carriers is 14.4% lower than that of CGC haplotype carriers 3 months after renal transplantation (Mao et al., 2021).

We identified other important variables in addition to CGC. The daily dose of CsA was positively associated with concentration, consistent with a previous study finding (Mao et al., 2020). In population analysis, the function of daily dose may primarily reflect the non-linearity of clearance, as seen in CsA concentration (Cai et al., 2020; Huang et al., 2020; Mao et al., 2020). In addition, the incorporation of the daily dose can significantly improve the model predictability. However, the depth of the relationship between CsA daily dose and concentration cannot be explored in the ML model.

Several biomedical indices, including WBC, HCT, PLT, TBIL, rGT, UN, CR, and CLCR, were also selected as important covariates of CsA blood concentration. Specifically, we reported that WBC, HCT, and PLT were positively correlated with CsA blood concentration. The relationship between the WBC count and CsA concentration has rarely been reported. For renal transplant patients, an elevated WBC count indicates potential infection or immune rejection. A higher dosage may increase CsA blood concentration, resulting in over-immunosuppression and subsequently infection.

Unlike WBC, the relationship between HCT and CsA concentration is commonly observed. In our previous study, the CL/*F* of CsA decreased significantly (52.6%) as HCT increased from 10.5% to 60.5% (Mao et al., 2021). Similarly, HCT was also selected as significant covariate inversely associated with tacrolimus CL/*F* (Woillard et al., 2011). A low HCT level may reduce the binding of CsA to red blood cells, increasing the proportion of CsA in plasma. Specifically, plasma CsA is easily metabolized, leading to a lower CsA blood concentration.

Meanwhile, Suehiro et al. (2002) found that both CsA and tacrolimus enhance platelet aggregation *via* the serotonin pathway. According to another study, CsA potentiates a collagen-evoked platelet procoagulant response (Tomasiak et al., 2007). Therefore, the level of PLT may indicate the level of CsA blood concentration. Additionally, TBIL and rGT levels, which reflect liver function, were positively associated with CsA blood concentration. Caban *et al.* observed that CsA could increase the levels of aspartate aminotransferase, alanine transaminase, and bilirubin by changing oxidative stress parameters and lipid peroxidation products in liver supernatants (Korolczuk et al., 2016). Changes in oxidative stress markers in parallel with mitochondrial damage suggest that the mechanisms play a crucial role in CsA-induced hepatotoxicity (Korolczuk et al., 2016). Poor liver function affects CsA metabolism, leading to a higher concentration of CsA.

Height was rarely identified as an influencing factor for CsA pharmacokinetics. However, it showed an inverse correlation with the CsA concentration. Sam *et al.* identified height as a significant influencing factor for the apparent volume of distribution in Asian liver transplant patients taking tacrolimus (Sam et al., 2006). They found that every meter of increase in height is associated with an 82.5% increase in V_d_/*F*. Based on other ML study on tacrolimus, height is also an important factor in the prediction model (Zheng et al., 2021).

POD after renal transplantation was also an influencing factor in CsA pharmacokinetics. Using the LASSO algorithm, in this study, we found that POD was negatively associated with CsA concentration. Likewise, Okada *et al.* and Mao *et al.* found that an increase in CL/*F*, along with POD, decreases the concentration of CsA (Okada et al., 2017; Mao et al., 2021).

As an immunosuppressive agent, CsA decreases the incidence of acute rejection and increases long-term survival after renal transplantation (Rodicio, 2000). Unfortunately, long-term CsA treatment can lead to several serious side effects, including systemic hypertension, permanent renal damage, cardiovascular disease, and numerous metabolic abnormalities. Calcium channel blockers (CCBs) are considered the best treatment for CsA-induced hypertension (Bernard et al., 2014). Certain CCBs, such as amlodipine, diltiazem, felodipine nicardipine, nifedipine, and verapamil, are relatively potent cytochrome P450 3A4 enzyme (CYP3A4) inhibitors at clinically relevant doses (Wang et al., 2016); these are metabolized by CYP3A4. In turn, these inhibit CYP3A4, which plays a key role in CsA metabolism (Bernard et al., 2014). As such, the co-administration of CCBs and CsA may increase CsA blood concentration. In our study, amlodipine, the first oral CCB used, was positively associated with CsA concentration. Bernard et al. (2014) found that blood trough concentrations of dose-normalized CsA increase significantly in patients treated with amlodipine, consistent with the results of our study . The co-prescription of acyclovir and CsA was also selected as a variable for increased CsA blood concentration.

Among the ML models, the ANN model exhibited the best predictive performance. Without *prior* observations, the predictive performance of the most suitable ML model was superior to that of the popPK model. The replacement of the popPK model with the ML model may depend on the model application scenario. ML algorithms could learn the hidden patterns from data themselves and do not require any *prior* knowledge. Therefore, for patients with unstable status or during initial dose optimization, the ML model is preferred (Woillard et al., 2021c). In addition, as ML models are data-driven, increasing the input participant data can continually optimize the parameters to improve accuracy and practicality. Therefore, the ML model is suitable for big-data analysis (observations >1000 and dimensions >50) without mechanistic assumptions (Graaf, 2014; McComb et al., 2021). However, the ML model works as a “black box,” and user-friendly interfaces should be developed to facilitate clinical application.

Moreover, the goals and possibilities of the ML and popPK models are different. No simulations were possible for the ML model, whereas the popPK model can simulate a time-concentration profile and estimate the probability of target achievement. Besides, popPK model is more flexible in regard to deviations in the sampling time. Specifically, the goal of the ML model is accuracy-centered, using the necessary variables. In contrast, the goal of the popPK model is to describe physiological phenomena and variability during the PK process. In addition, the mathematics underlying each method is different. To increase model predictability and interpretability, a combination of ML and pharmacometrics models may be necessary (Koch et al., 2020; Tang et al., 2021).

Among all patients recruited in this study, the percentages of follow-up concentrations within the target CsA C_0_ and C_2_ were 46.2% and 39.0%, respectively. This result highlighted the need to perform model-informed precision dosing in clinical practice (Kluwe et al., 2020). According to our results, the predictability and suggested dose regimens of the ML and popPK models were comparable. However, there is also some discrepancy between these two methods. The final prescription should be determined by the combination of the predicted dosage and clinical information.

This study had limitations. First, we used a retrospective, observational design. Therefore, the adherence of patients to their prescribed dose regimens cannot be confirmed. Second, the TDM data used were collected from one center. Therefore, multicenter validation is necessary to confirm the model predictability. Third, we applied covariates selection using a linear association-based method, and then used those covariates for nonlinear model construction. This procedure might remove features that could have been of interest. Fourth, the distribution of CsA concentrations was not equivalent. Approximately only 15% of cases had a CsA concentration above 1200 ng/ml, and ML algorithms have difficulty extracting useful information from limited samples with high CsA concentrations. The ML model should be used with caution to predict concentrations higher than 1200 ng/ml. Finally, the relationship between the dependent and independent variables was extremely complicated in all statistical algorithms, and the existence of gene-gene and gene-environment interactions introduces more challenges for researchers (Hunter, 2005).

## 5 Conclusion

The predictability of the ML and popPK models was comparable, except for linear regression, indicating that considering the nonlinear relationship of patient covariates may help to improve the model predictability. These results could facilitate the application of ML models in clinic, especially for patients with unstable status or during initial dose optimization.

## Data Availability

The raw data supporting the conclusion of this article will bemade available by the authors, without undue reservation.
