# Supplementary figures and images for: Applying machine learning to the pharmacokinetic modeling of cyclosporine in adult renal transplant recipients: a multi-method comparison
Source: Front Pharmacol. 2022 Oct 24;13:1016399. doi: 10.3389/fphar.2022.1016399 (PMC9664902; doi:10.3389/fphar.2022.1016399)

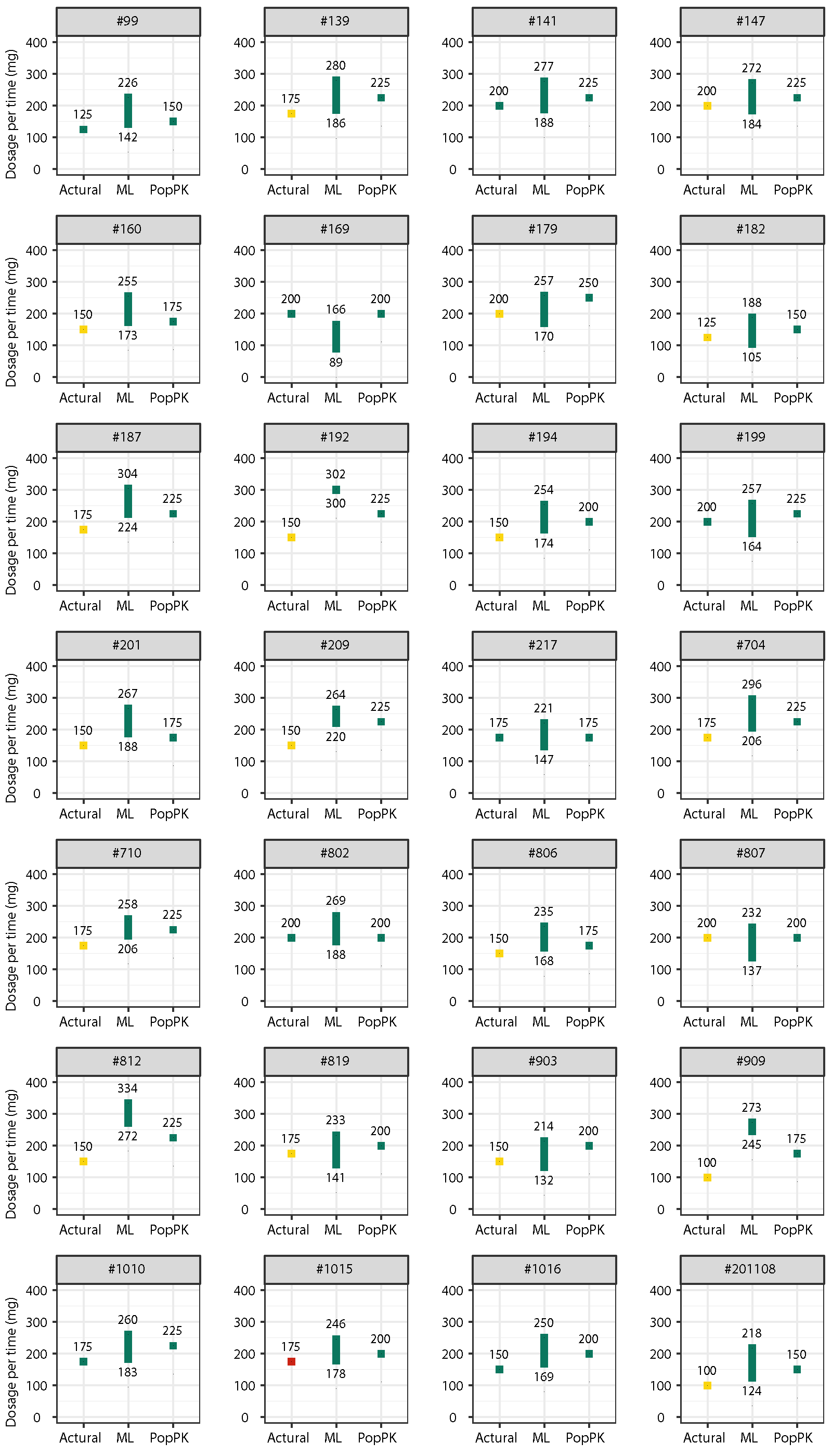

Supplement: Supplementary file 1 [file Image1.TIFF]
