# Supplementary material for: Applying machine learning to the pharmacokinetic modeling of cyclosporine in adult renal transplant recipients: a multi-method comparison
Source: Front Pharmacol. 2022 Oct 24;13:1016399. doi: 10.3389/fphar.2022.1016399 (PMC9664902; doi:10.3389/fphar.2022.1016399)
Supplement: Supplementary file 2 [file DataSheet2.docx]

# Electronic Supplementary Material

## Supplementary Text S1 Immunosuppressive therapeutic regimen

All patients were administered combined immunosuppressive therapy, including a CsA microemulsion (Neoral; Novartis Pharma Schweiz AG, Emberbach, Germany), mycophenolate mofetil (MMF; CellCept; Roche Pharma Ltd., Shanghai, China) and corticosteroids. The initial dose of CsA was 5 mg kg^-1^ day^-1^, administered as two doses under fasting conditions immediately after surgery. Subsequent doses were empirically adjusted to achieve target concentrations based on local guidelines [[1](#_ENREF_1)]. MMF (0.5 - 3 g day^-1^) was administered according to body size and POD. This schedule was followed with oral prednisolone (80 mg day^-1^), and the dosage was gradually decreased by 10 mg day^-1^ until reaching 20 mg d^-1^ after 10 d. The dosage was further tapered to 15, 10, and 5 mg day^-1^ by months 1, 3, and 6, respectively.

## Supplementary Text S2 Determination of the CsA concentration

The samples were collected from May 2011 to December 2016 and analyzed by chemiluminescent microparticle immunoassay (CMIA) on an Architect I2000 system (Abbott Diagnostics, Chicago, IL, USA). The remaining samples were analyzed by a well-validated fluorescence polarization immunoassay (FPIA) on an AxSYM Abbott diagnostic system (Abbott Diagnostics, Chicago, IL, USA).

Due to the systematic biases and the cross-reactivity of metabolites between the methods, the following equation (Eq. 1) [[2](#_ENREF_2)] was employed to convert the CMIA-measured C_0_ to that of FPIA.

$AxSYM =0.87\times CMIA+25.84$ (1) [[2](#_ENREF_2)]

Here, AxSYM represents the FPIA performed using an AxSYM analyzer, whereas CMIA represents the CsA concentration detected using an Architect system. For AxSYM, the limit of detection (LOD) was 21.8 ng mL^-1^, and the calibration range was 40 - 800 ng mL^-1^; for CMIA, the LOD was 25 ng mL^-1^, and the calibration range was 30 - 1500 ng mL^-1^.

## Supplementary Text S3 Genotyping and haplotype analysis of CYP3A4*1G, CYP3A5*3, ABCB1 C1236T, G2677T/A, and C3435T single-nucleotide polymorphisms

The genotyping of *CYP3A4*1G, CYP3A5*3, ABCB1* C1236T, G2677T/A, and C3435T was performed by an independent external contractor (GeneCore BioTechnologies Co., Ltd., Shanghai, China) using a DNA sequencing apparatus (Applied Biosystems 3730; Thermo Fisher Scientific, Waltham, MA, USA) [[3](#_ENREF_3)].

Genomic DNA was extracted from whole blood using TIANamp Blood DNA Kit (TIANGEN, DP318). Polymerase chain reaction (PCR) was performed in a reaction volume of 20μL, containing 10μL MIX, 7μLddH_2_O, 1μL forward primer, 1μL backward primer and 1μL DNA template. The sequences of forward and reverse primers were listed below.

**The sequences of primers for genotyping SNPs of CYP3A5 and ABCB1**

| SNP | Primer |
| --- | --- |
| CYP3A4*1G  (rs2242480) | F: 5’CACCCTGATGTCCAGCAGAAACT3’ |
|  | R: 5’AATAGAAAGCAGATGAACCAGAGCC3’ |
| CYP3A5*3  (rs776746) | Forward: 5’CTTTAAAGAGCTCTTTTGTCTCTC3’ |
|  | Reverse: 5’CCACGAAGCCAGACTTTGAT3’ |
| ABCB1 C1236T  (rs1128503) | Forward: 5’TCTTTGTCACTTTATCCAGC3’ |
|  | Reverse: 5’TCTCACCATCCCCTCTGT3’ |
| ABCB1 G2677T/A  (rs2032582) | Forward: 5’TGCAGGCTATAGGTTCCAGG3’ |
|  | Reverse: 5’TTTAGTTTGACTCACCTTCCCG3’ |
| ABCB1 C3435T  (rs1045642) | Forward: 5’TGCTGGTCCTGAAGTTGATCTGTGAAC3’ |
|  | Reverse:5’ACATTAGGCAGTGACTCGATGAAGGCA3’ |

Cycle conditions were: initial denaturation at 95°C for 5 min, then 35 cycles of denaturation at 95°C for 15s, annealing at 55°C for 15s and elongation at 72°C for 30s, followed by a final extension at 72°C for 5 min. PCR products were analyzed by electrophoresis with 2% agarose gels (DNA Marker DL2000) and purified using PCR Product Purification Kit (Bodataike Bioengineering Corporation, Peiking, China).

Deviations from the Hardy-Weinberg equilibrium were tested using Pearson’s χ^2^-test. The absolute standardized linkage disequilibrium (LD) coefficient was used to determine the LD between different pairs of *ABCB1* single-nucleotide polymorphisms (SNPs). The analysis was like that of a previous report [[4](#_ENREF_4)].

## Supplementary Table S1 The integrated list of variables abbreviations and corresponding explanations

| Covariate abbreviation | Explanation |
| --- | --- |
| ID | identity number |
| TIME | time at sample collection |
| DOSE1 | the penultimate CsA dosage before sample collection |
| DOSE2 | the latest CsA dosage before sample collection |
| DV | CsA blood concentration |
| TAD | Sample collecting time |
| HT | Height |
| BMI | body mass index |
| BSA | body surface area |
| FFM | fat free mass |
| Sour | source of transplanted renal |
| POD | postoperative days |
| DD | daily dose |
| AGE | Age |
| GEND | Gender |
| WT | Weight |
| FORM | formation |
| WBC | white blood cell count |
| RBC | red blood cell count |
| HCT | hematocrit |
| NEUT | neutrophil |
| PLT | platelet count |
| ALT | alanine amino transferase |
| AST | Aspartate aminotransferase |
| TBIL | total bilirubin |
| ALP | alkaline phosphatase |
| RGT | r-Glutamyl transpeptidase |
| ALB | albumin |
| GLB | Globin |
| TP | total protein |
| UN | urea nitrogen |
| CR | serum creatinine |
| CLCR | creatinine clearance |
| UA | uric acid |
| CGC | MDR1 haplotype |
| DMMF | mycophenolate mofetil dosage |
| MIZO | mizoribine dosage |
| MF | myfortic dosage |
| PD | prednisone dosage |
| MP | methylprednisolone dosage |
| JXZ | metronidazole |
| BYD | felodipine |
| BTLK | metoprolol |
| ACI | acyclovir |
| SN | furosemide |
| BXT | nifedipine |
| AK | omeprazole |
| NOR | norvasc |
| PEDP | perdipine |
| LNTD | ranitidine |
| KLBT | levofloxacin |
| LZPL | azathioprine |

## Supplementary Table S2 Allele frequencies of genetic polymorphisms in CYP3A4, CYP3A5 and ABCB1 genes

| **Single nucleotide polymorphisms** | **Number of recipients** | **Frequency (%)** |
| --- | --- | --- |
| CYP3A4*1G (G82266A, rs 2242480) |  |  |
| GG (*1/*1) | 95 | 56.9 |
| GA (*1/*1G) | 63 | 37.7 |
| AA (*1G/*1G) | 9 | 5.4 |
| CYP3A5*3 (A6986G, rs776746) |  |  |
| AA (*1/*1) | 8 | 4.8 |
| GA (*1/*3) | 75 | 44.9 |
| GG (*3/*3) | 84 | 50.3 |
| ABCB1-C1236T (rs1128503) |  |  |
| CC | 25 | 15.0 |
| CT | 68 | 40.7 |
| TT | 74 | 44.3 |
| ABCB1-G2677T/A (rs2032582) |  |  |
| AA | 9 | 5.4 |
| GG | 40 | 24.0 |
| GA | 20 | 12.0 |
| TT | 31 | 18.6 |
| TG | 54 | 32.3 |
| TA | 13 | 7.8 |
| ABCB1-C3435T (rs1045642) |  |  |
| CC | 63 | 37.7 |
| CT | 79 | 47.3 |
| TT | 25 | 15.0 |

The allele frequencies are found to be in Hardy-Weinberg equilibrium (*P* > 0.05)

## Supplementary Table S3 Determination of ABCB1 C1236T-G2677T/A-C3435T haplotype with frequency and patient proportion more than 8%

| Haplotype | C1236T | G2677T/A | C3435T | Number | Total number | Proportion (%) |
| --- | --- | --- | --- | --- | --- | --- |
| TTT | TT | TT | TT | 18 | 31 | 18.56 |
|  | TT | TT | CT | 9 |  |  |
|  | TT | GT | TT | 2 |  |  |
|  | TT | TA | TT | 0 |  |  |
|  | CT | TT | TT | 2 |  |  |
| TGC | TT | GG | CC | 11 | 28 | 16.77 |
|  | TT | GG | CT | 3 |  |  |
|  | CT | GG | CC | 8 |  |  |
|  | TT | GT | CC | 6 |  |  |
|  | TT | GA | CC | 0 |  |  |
| CGC | CC | GA | CC | 8 | 25 | 14.97 |
|  | CC | GG | CC | 7 |  |  |
|  | CC | GG | CT | 2 |  |  |
|  | CT | GG | CC | 8 |  |  |
|  | CC | GT | CC | 0 |  |  |
| CAC | CC | AA | CC | 6 | 16 | 9.58 |
|  | CC | AA | CT | 1 |  |  |
|  | CT | AA | CC | 1 |  |  |
|  | CC | GA | CC | 8 |  |  |
|  | CC | TA | CC | 0 |  |  |

## Supplementary Table S4 Coefficients of selected covariates by LASSO model with minimal prediction error

| **No.** | **Covariate** | **Coefficient** |
| --- | --- | --- |
| 1 | DOSE1 | 2.11×10^-3^ |
| 2 | DOSE2 | 1.64×10^-3^ |
| 3 | TAD | 1.54 |
| 4 | HT | -0.361 |
| 5 | POD | -1.73×10^-4^ |
| 6 | WBC | 2.97×10^-4^ |
| 7 | HCT | 8.69×10^-5^ |
| 8 | PLT | 2.36×10^-4^ |
| 9 | TBIL | 2.31×10^-4^ |
| 10 | RGT | 1.51×10^-4^ |
| 11 | UN | -3.32×10^-3^ |
| 12 | CR | -2.12×10^-4^ |
| 13 | CLCR | 1.89×10^-4^ |
| 14 | CGC | 6.19×10^-2^ |
| 15 | ACI | 8.25×10^-2^ |
| 16 | NOR | 3.05×10^-2^ |

ACI, acyclovir; CGC, MDR1 haplotype; CLCR, creatinine clearance; CR, serum creatinine; DOSE1, the penultimate cyclosporine dosage before sample collection; DOSE2, the latest cyclosporine dosage before sample collection; RGT, r-Glutamyl transpeptidase; HCT, hematocrit; HT, height; NOR, Norvasc; PLT, alanine amino transferase; POD, postoperative days; TAD, Sample collecting time; TBIL, total bilirubin; UN, urea nitrogen; WBC, white blood cell count

## Supplementary Table S5 The best-tuned parameters for ML models

| Model | Parameter | Value |
| --- | --- | --- |
| SVR |  |  |
|  | Cost | 1 |
|  | epsilon | 0.01 |
|  | gamma | 0.1 |
| Random Forest |  |  |
|  | max_depth | 6 |
|  | min_samples_leaf | 12 |
|  | min_samples_split | 50 |
|  | n_estimators | 90 |
| XGBoost |  |  |
|  | colsample_bytree | 0.4 |
|  | gamma | 0.1 |
|  | learning_rate | 0.05 |
|  | max_depth | 4 |
|  | min_child_weight | 7 |
|  | n_estimators | 300 |
| LightGBM |  |  |
|  | max_depth | 10 |
|  | min_data_in_leaf | 25 |
|  | num_leaves | 7 |
| CatBoost |  |  |
|  | depth | 4 |
|  | iterations | 800 |
|  | l2_leaf_reg | 9 |
|  | learning_rate | 0.05 |
|  | thread_count | 4 |
| ANN |  |  |
|  | batch size | 32 |
|  | num_hidden_layer | 2 |
|  | num_neuron_ hidden_layer1 | 40 |
|  | num_neuron_ hidden_layer2 | 25 |
|  | activation_function_ hidden_layer | tanh |
|  | dropout_ hidden_layer1 | 0.2 |
|  | dropout_ hidden_layer2 | 0.05 |
|  | activation_function_ output_layer | relu |
|  | optimizer | adam |

ANN, artificial neural network; ML, machine learning; SVR, support vector regression

## Supplementary Table S6 Comparison of actual and optimal CsA dose regimens recommended by the most suitable ML and popPK models

| ID | Actual C_0_  (ng mL^-1^) | Actual C_2_  (ng mL^-1^) | Actual DOSE1 (mg) | Actual DOSE2  (mg) | Lower limit  recommended by ML (mg)^a^ | Upper limit  recommended by ML (mg)^a^ | Dosage regimens recommended by popPK (mg)^a^ |
| --- | --- | --- | --- | --- | --- | --- | --- |
| 99 | 288.9 | 1261.9 | 125 | 125 | 142 | 226 | 150 |
| 139 | 160.1 | 804 | 175 | 175 | 186 | 280 | 225 |
| 141 | 226.4 | 1479 | 200 | 200 | 188 | 277 | 225 |
| 147 | 162.9 | 887.2 | 200 | 200 | 184 | 272 | 225 |
| 160 | 200.6 | 567.2 | 150 | 150 | 173 | 255 | 175 |
| 169 | 223.8 | 1509.4 | 200 | 200 | 89 | 166 | 200 |
| 179 | 234.6 | 699.6 | 200 | 200 | 170 | 257 | 250 |
| 182 | 137.7 | 736.4 | 125 | 125 | 105 | 188 | 150 |
| 187 | 132.7 | 704.4 | 175 | 175 | 224 | 304 | 225 |
| 192 | 161.5 | 436.8 | 150 | 150 | 300 | 302 | 225 |
| 194 | 122.3 | 644 | 150 | 150 | 174 | 254 | 200 |
| 199 | 272.1 | 1105.6 | 200 | 200 | 164 | 257 | 225 |
| 201 | 123 | 888 | 150 | 150 | 188 | 267 | 175 |
| 209 | 75 | 942.8 | 150 | 150 | 220 | 264 | 225 |
| 217 | 271.2 | 1243.2 | 175 | 175 | 147 | 221 | 175 |
| 704 | 121.3 | 830.8 | 175 | 175 | 206 | 296 | 225 |
| 710 | 108.7 | 785.2 | 175 | 175 | 206 | 258 | 225 |
| 802 | 200 | 1453.8 | 200 | 200 | 188 | 269 | 200 |
| 806 | 183.6 | 853.6 | 150 | 150 | 168 | 235 | 175 |
| 807 | 306.6 | 932.4 | 200 | 200 | 137 | 232 | 200 |
| 812 | 193.1 | 586.6 | 150 | 150 | 272 | 334 | 225 |
| 819 | 217.5 | 975 | 175 | 175 | 141 | 233 | 200 |
| 903 | 118.9 | 883.8 | 150 | 150 | 132 | 214 | 200 |
| 909 | 141.4 | 533.2 | 100 | 100 | 245 | 273 | 175 |
| 1010 | 279.9 | 1145.2 | 175 | 175 | 183 | 260 | 225 |
| 1015 | 418.1 | 1058.6 | 175 | 175 | 178 | 246 | 200 |
| 1016 | 225.9 | 1366.2 | 150 | 150 | 169 | 250 | 200 |
| 201108 | 160.7 | 941.3 | 100 | 100 | 124 | 218 | 150 |

C_0_, pre-dose concentration; C_2_, 2-hour post-dose concentration; ML, machine learning; popPK, population pharmacokinetic model

^a^ All the doses are recommended twice daily

Five patients were highlighted as the dosage suggested by the ML model and popPK model were inconsistent with each other.

## Supplementary Table S7 Dose regimens recommended by population pharmacokinetic model

| Simulation  scheme | Dose regimen  recommended | C_0_^a^ | C_2_^a^ |
| --- | --- | --- | --- |
| #99-1 | 125 mg bid | 193.3 (124.8-289.5) | 944.9 (696.8-1239.1) |
| #99-2 | 150 mg bid | 232.0 (149.7-347.4) | 1133.9 (836.2-1486.9) |
| #99-3 | 175 mg bid | 270.6 (174.7-405.3) | 1322.9 (975.5-1734.7) |
| #139-1 | 200 mg bid | 214.6 (142.3-312.1) | 903.0 (665.5-1184.9) |
| #139-2 | 225 mg bid | 241.4 (160.1-351.1) | 1015.9 (748.7-1333.0) |
| #139-3 | 250 mg bid | 268.3 (177.9-390.1) | 1128.8 (831.9-1481.1) |
| #141-1 | 200 mg bid | 221.6 (142.6-327.8) | 977.7 (719.2-1269.7) |
| #141-2 | 225 mg bid | 249.3 (160.4-368.7) | 1099.9 (809.1-1428.4） |
| #141-3 | 250 mg bid | 277.0 (178.3-409.7) | 1222.1 (899.0-1587.1) |
| #147-1 | 200 mg bid | 214.3 (137.8-316.9) | 954.3 (703.4-1240.8) |
| #147-2 | 225 mg bid | 241.1 (155.1-356.5) | 1073.6 (791.3-1395.9) |
| #147-3 | 250 mg bid | 267.9 (172.3-396.1) | 1192.9 (879.3-1551.0) |
| #160-1 | 150 mg bid | 205.6 (131.2-308.3) | 1053.4 (777.4-1383.5) |
| #160-2 | 175 mg bid | 239.8 (153.1-359.7) | 1228.9 (906.9-1614.1) |
| #160-3 | 200 mg bid | 274.1 (175.0-411.1) | 1404.5 (1036.5-1844.6) |
| #169-1 | 175 mg bid | 255.3 (165.3-371.8) | 931.5 (692.9-1218.9) |
| #169-2 | 200 mg bid | 291.8 (188.9-424.9) | 1064.6 (791.8-1393.1) |
| #169-3 | 225 mg bid | 328.3 (212.5-478.0) | 1197.7 (890.8-1567.2) |
| #179-1 | 225 mg bid | 205.6 (134.3-300.5) | 970.6 (716.9-1269.3) |
| #179-2 | 250 mg bid | 226.6 (148.0-330.7) | 1075.6 (796.2-1405.9) |
| #179-3 | 275 mg bid | 247.9 (161.4-362.7) | 1180.5 (873.6-1545.4) |
| #182-1 | 125 mg bid | 217.9 (142.0-324.9) | 981.0 (723.1-1283.4) |
| #182-2 | 150 mg bid | 261.5 (170.5-289.8) | 1177.2 (867.7-1540.1) |
| #182-3 | 175 mg bid | 305.1 (198.9-454.8) | 1373.4 (1012.4-1796.8) |
| #187-1 | 200 mg bid | 212.3 (137.6-315.8) | 971.1 (716.6-1267.7) |
| #187-2 | 225 mg bid | 238.8 (154.8-355.3) | 1092.6 (806.2-1426.2) |
| #187-3 | 250 mg bid | 265.3 (172.0-394.8) | 1213.9 (895.7-1584.7) |
| #192-1 | 200 mg bid | 215.3 (140.7-318.0) | 1003.5 (750.8-1316.3) |
| #192-2 | 225 mg bid | 242.2 (158.3-357.8) | 1128.9 (844.6-1480.9) |
| #192-3 | 250 mg bid | 269.1 (175.8-397.5) | 1254.3 (938.5-1645.4) |
| #194-1 | 175 mg bid | 222.4 (144.7-328.8) | 948.4 (698.4-1225.9) |
| #194-2 | 200 mg bid | 254.1 (165.4-375.8) | 1084.0 (798.2-1401.0) |
| #194-3 | 225 mg bid | 285.9 (186.1-422.7) | 1219.4 (898.0-1576.0) |
| #199-1 | 200 mg bid | 227.6 (147.3-335.0) | 922.0 (679.5-1193.0) |
| #199-2 | 225 mg bid | 256.1 (165.7-376.9) | 1037.2 (764.4-1342.2) |
| #199-3 | 250 mg bid | 284.5 (184.1-418.7) | 1152.5 (849.3-1491.3) |
| #201-1 | 150 mg bid | 187.4 (104.6-284.0) | 1040.3 (789.9-1383.6) |
| #201-2 | 175 mg bid | 212.9 (121.0-321.1) | 1207.5 (918.4-1605.6) |
| #201-3 | 200 mg bid | 238.8 (137.1-263.1) | 1375.6 (1042.5-1828.5) |
| #209-1 | 200 mg bid | 185.7 (118.2-283.7) | 1000.6 (737.3-1310.6) |
| #209-2 | 225 mg bid | 209.0 (133.0-319.1) | 1125.7 (829.5-1474.5) |
| #209-3 | 250 mg bid | 232.2 (147.8-354.6) | 1250.8 (921.6-1638.3) |
| #217-1 | 150 mg bid | 215.8 (145.5-314.9) | 868.6 (645.9-1143.5) |
| #217-2 | 175 mg bid | 251.7 (169.8-367.3) | 1013.4 (753.6-1334.0) |
| #217-3 | 200 mg bid | 287.7 (194.1-419.8) | 1158.1 (861.2-1524.7) |
| #704-1 | 200 mg bid | 233.2 (150.4-344.7) | 1029.9 (758.9-1339.1) |
| #704-2 | 225 mg bid | 262.4 (169.2-387.8) | 1158.6 (853.8-1506.5) |
| #704-3 | 250 mg bid | 291.5 (188.0-430.9) | 1287.3 (948.6-1673.9) |
| #710-1 | 200 mg bid | 234.9 (156.1-342.3) | 992.1 (733.0-1303.3) |
| #710-2 | 225 mg bid | 264.3 (175.6-385.1) | 1116.2 (824.7-1466.2) |
| #710-3 | 250 mg bid | 293.6 (195.1-427.9) | 1240.2 (916.3-1629.1) |
| #802-1 | 175 mg bid | 231.6 (151.0-335.2) | 1049.2 (779.0-1378.7) |
| #802-2 | 200 mg bid | 258.4 (170.1-378.3) | 1193.0 (886.5-1572.2) |
| #802-3 | 225 mg bid | 287.3 (188.8-420.5) | 1340.1 (994.2-1762.1) |
| #806-1 | 150 mg bid | 237.2 (153.9-350.1) | 1104.2 (812.2-1442.5) |
| #806-2 | 175 mg bid | 276.7 (179.6-408.4) | 1288.2 (947.6-1682.9) |
| #806-3 | 200 mg bid | 316.3 (205.3-466.8) | 1472.2 (1083-1923.3) |
| #807-1 | 175 mg bid | 201.3 (129.6-297.3) | 891.3 (656.2-1160.3) |
| #807-2 | 200 mg bid | 230.0 (148.1-339.7) | 1018.7 (750.0-1326.2) |
| #807-3 | 225 mg bid | 268.7 (177.9-388.1) | 1147.1 (851.4-1533.8) |
| #812-1 | 200 mg bid | 206.0 (133.2-319.1) | 986.8 (729.5-1323.9) |
| #812-2 | 225 mg bid | 231.7 (149.8-359.0) | 1110.1 (820.7-1489.4) |
| #812-3 | 250 mg bid | 257.5 (166.5-398.8) | 1233.5 (911.9-1654.9) |
| #819-1 | 175 mg bid | 229.4 (153.8-336.7) | 944.4 (702.5-1243.3) |
| #819-2 | 200 mg bid | 262.2 (175.7-384.8) | 1079.4 (802.9-1421.0) |
| #819-3 | 225 mg bid | 294.9 (197.7-432.9) | 1214.2 (903.2-1598.6) |
| #903-1 | 175 mg bid | 175.6 (108.6-280.1) | 973.8 (725.3-1307.8) |
| #903-2 | 200 mg bid | 200.7 (124.2-320.1) | 1112.9 (828.9-1494.7) |
| #903-3 | 225 mg bid | 225.7 (139.7-360.1) | 1252.0 (932.6-1681.5) |
| #909-1 | 150 mg bid | 192.8 (123.1-283.5) | 880.7 (660.8-1155.4) |
| #909-2 | 175 mg bid | 225.0 (143.6-330.8) | 1027.5 (771.0-1348.0) |
| #909-3 | 200 mg bid | 257.1 (164.2-378.0) | 1174.3 (881.1-1540.6) |
| #1010-1 | 200 mg bid | 216.7 (142.6-324.1) | 1027.4 (762.3-1361.0) |
| #1010-2 | 225 mg bid | 243.8 (160.4-364.6) | 1155.8 (857.6-1531.1) |
| #1010-3 | 250 mg bid | 270.9 (178.3-405.2) | 1284.3 (952.9-1701.1) |
| #1015-1 | 175 mg bid | 216.9 (141.2-318.0) | 923.5 (681.5-1207.5) |
| #1015-2 | 200 mg bid | 247.9 (161.4-363.5) | 1055.5 (778.9-1379.9) |
| #1015-3 | 225 mg bid | 278.9 (181.5-408.9) | 1187.4 (876.2-1552.4) |
| #1016-1 | 175 mg bid | 196.4 (128.5-286.8) | 892.7 (633.6-1178.5) |
| #1016-2 | 200 mg bid | 221.1 (144.9-322.4) | 1018.6 (755.1-1338.4) |
| #1016-3 | 225 mg bid | 245.6 (160.7-356.3) | 1140.6 (847.5-1500.3) |
| #201108-1 | 125 mg bid | 207.3 (132.8-309.6) | 1027.8 (761.4-1344.0) |
| #201108-2 | 150 mg bid | 244.0 (156.3-366.5) | 1229.4 (913.7-1606.4) |
| #201108-3 | 175 mg bid | 281.5 (181.4-424.0) | 1427.7 (1064.0-1870.5) |

C_0_, pre-dose concentration; C_2_, 2-hour post-dose concentration

^a^ Data are expressed as median (25% - 75% percentiles)

1. Shi BY, Yuan M. Guidelines for immunosuppressive therapy in Chinese renal transplant recipients. Organ Transplantation 2016; 7: 327-31.

2. Serdarevic N, Zunic L. Comparison of architect I 2000 for determination of cyclosporine with axsym. Acta informatica medica : AIM : journal of the Society for Medical Informatics of Bosnia & Herzegovina : casopis Drustva za medicinsku informatiku BiH 2012; 20: 214-7.

3. Geng F, Jiao Z, Dao YJ, Qiu XY, Ding JJ, Shi XJ, Li ZD, Zhong MK. The association of the UGT1A8, SLCO1B3 and ABCC2/ABCG2 genetic polymorphisms with the pharmacokinetics of mycophenolic acid and its phenolic glucuronide metabolite in Chinese individuals. Clinica chimica acta; international journal of clinical chemistry 2012; 413: 683-90.

4. Mao J, Qiu X, Qin W, Xu L, Zhang M, Zhong M. Factors Affecting Time-Varying Clearance of Cyclosporine in Adult Renal Transplant Recipients: A Population Pharmacokinetic Perspective. Pharmaceutical research 2021; 38: 1873-87.
