# Supplementary material for: Applying machine learning to the pharmacokinetic modeling of cyclosporine in adult renal transplant recipients: a multi-method comparison
Source: Front Pharmacol. 2022 Oct 24;13:1016399. doi: 10.3389/fphar.2022.1016399 (PMC9664902; doi:10.3389/fphar.2022.1016399)
Supplement: Supplementary file 3 [file DataSheet1.docx]

# Supporting information

Additional Supporting Information may be found in the online version of this article at the publisher’s web-site:

***Supplementary Text S1*** Immunosuppressive therapeutic regimen

***Supplementary Text S2*** Determination of the CsA concentration

***Supplementary Text S3*** Genotyping and haplotype analysis of CYP3A4*1G, CYP3A5*3, ABCB1 C1236T, G2677T/A, and C3435T single-nucleotide polymorphisms

***Table S1*** The integrated list of variables abbreviations and corresponding explanations

***Table S2*** Allele frequencies of genetic polymorphisms in CYP3A4, CYP3A5 and ABCB1 genes

***Table S3*** Determination of ABCB1 C1236T-G2677T/A-C3435T haplotype with frequency and patient proportion more than 8%

***Table S4*** Coefficients of selected covariates by LASSO model with minimal prediction error

***Table S5*** The best-tuned parameters for ML models

***Table S6*** Comparison of actual and optimal cyclosporine dosage regimens recommended by the most suitable ML and popPK models

***Table S7*** Dose regimens recommended by population pharmacokinetic model

***Figure S1*** Comparison of the actual and optimal daily doses of cyclosporine recommended by the most suitable ML and popPK models for twenty-eight patients. Yellow plot indicates the concentration below the therapeutic windows; green plot indicates the concentration in the therapeutic windows; red plot indicates the concentration above the therapeutic windows. All the dosages are recommended twice daily. ML, machine learning; popPK, population pharmacokinetic model.
